# Optimized real-time path planning for micro UAVs in dynamic environments aided by reciprocal velocity obstacle algorithm

**DOI:** 10.1371/journal.pone.0336098

**Published:** 2025-11-17

**Authors:** Pengxiang Sun, Wei Sun, Wei Ding, Yadan Li, Jingang Zhao

**Affiliations:** School of Geomatics, Liaoning Technical University, Fuxin, China; Embry-Riddle Aeronautical University, UNITED STATES OF AMERICA

## Abstract

With the rapid deployment of autonomous micro-UAVs in dynamic environments, path planning must ensure both safety and real-time performance under stringent onboard computational constraints. This paper proposes a dynamic path planning method based on the reciprocal velocity obstacles algorithm, enabling micro-UAVs to safely and efficiently accomplish flight tasks in complex environments. In three-dimensional space, we introduce the Velocity-Obstacle Spherical Crown (VOSC) model to delineate safe and feasible velocity boundaries, thereby ensuring reliable avoidance of moving obstacles. Within this velocity domain, a minimum-deflection-angle replanning strategy generates smooth and dynamically feasible trajectories. For multi-obstacle scenarios, we design a critical-curve-based avoidance scheme that allows the UAV to flexibly select feasible maneuvers along the curve, improving efficiency and robustness. Simulation results demonstrate that, compared with traditional methods, the proposed approach significantly reduces planning time while enhancing trajectory smoothness. Moreover, the algorithm runs online on micro-UAV hardware, highlighting its potential for warehouse navigation, low-altitude urban transport, and other real-time missions.

## I. Introduction

With the rapid development of communication and control technologies, the autonomous control of intelligent aerial platforms, especially micro unmanned aerial vehicles (UAVs), has become a significant focus in the field of robotics due to their agility, adaptability, and wide application potential [1–3]. UAVs are capable of operating in environments that are otherwise inaccessible to humans, performing tasks such as surveillance, material transportation, and emergency rescue missions [4,5]. To ensure safe and efficient operation, UAV control systems must address two fundamental challenges: path generation and motion regulation. The former defines the feasible trajectory within a spatial environment, while the latter ensures that the UAV can follow the planned path by regulating parameters such as velocity and acceleration.

Classical path planning approaches, such as the artificial potential field (APF) method, A* algorithm, and Dijkstra’s algorithm, provide basic frameworks for route generation [6–9]. However, these methods suffer from significant limitations when applied in dynamic and complex environments. APF methods often fall into local minima, while graph-based search methods generate discontinuous trajectories, which are unsuitable for real-time UAV motion execution [10–12]. In response to these deficiencies, curve-based interpolation strategies such as polynomial fitting and Bézier or B-spline curves have been proposed to generate smooth and differentiable paths [13–15]. These techniques incorporate trajectory continuity and dynamic feasibility constraints but typically assume a static environment or require significant offline computation.

Recent efforts have focused on path optimization in dynamic environments, which introduces additional complexity due to time-varying obstacles and the need for predictive motion planning. Dynamic path planning must reconcile real-time environmental perception, obstacle motion prediction, and optimal trajectory adjustment, all under limited onboard computational resources [16–18]. The Reciprocal Velocity Obstacle (RVO) framework offers a promising paradigm by modeling reciprocal interactions between agents based on velocity space constraints. Jamie et al. achieved collision-free and oscillation-free navigation under conditions where agents independently compute their own solutions [19]. Han et al. combined the concept of Reciprocal Velocity Obstacles (RVO) with Deep Reinforcement Learning (DRL) to enhance multi-agent collision avoidance in crowded environments [20]. D. Alejo et al. considered the characteristics of quadrotor UAVs and realized rapid avoidance of dynamic obstacles in 3D space [21]. However, conventional RVO-based implementations struggle with trajectory smoothness and local convergence issues in cluttered environments, especially when integrated with high-order path representations.

To address these theoretical and practical limitations, this study proposes a dynamically adaptive path planning framework that integrates B-spline-based trajectory modeling with a real-time RVO algorithm, further enhanced by a novel obstacle threat quantification parameter and a deflection-based trajectory correction strategy. The main theoretical contributions of this work are as follows:

(1) Trajectory Modeling with Convex B-spline Curves: We introduce a parametric representation of the global trajectory using B-spline interpolation, allowing the system to encode high-order smoothness constraints and spatial controllability while preserving convex properties essential for dynamic replanning.
(2) Predictive Collision Avoidance with Velocity Obstacle Spatial Constraint (VOSC): We define a new metric that quantitatively models the influence region of moving obstacles in velocity space, grounded in reciprocal collision theory and spatial-temporal risk analysis, enabling proactive obstacle avoidance decisions.
(3) Minimum-Deflection Replanning Strategy: By incorporating a geometric minimization strategy for deviation angles, we propose a local trajectory correction method that guarantees real-time feasibility and smooth convergence to the original path after obstacle clearance.

The proposed framework contributes to the theoretical advancement of dynamic path planning by combining geometric modeling, motion prediction, and convex optimization strategies into a unified control architecture. Simulation results validate the framework’s performance in achieving collision-free, dynamically feasible, and computationally efficient path planning for micro UAVs operating in cluttered and dynamic environments.

**Fig 1:**
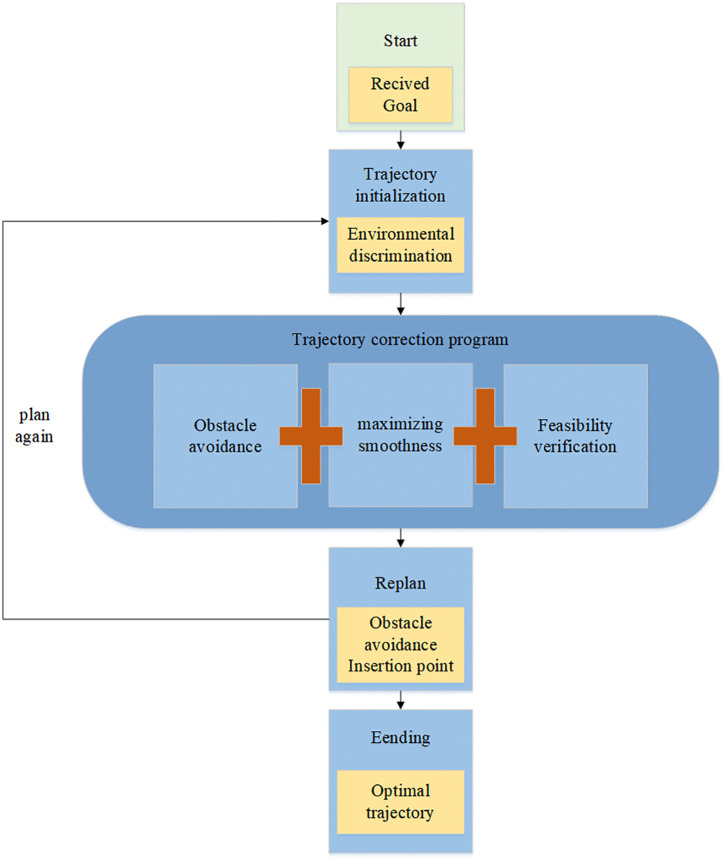
Flowchart for UAV trajectory planning.

The flowchart of UAV trajectory planning is shown in Fig 1.

The first section of this paper reviews the related work and the current state of development in autonomous UAV flight. The second section details the UAV trajectory initialization and establishment of VOSC. The third section illustrated how to use VOSC to solve obstacle avoidance direction, highlighting the key aspects of our approach. The fourth section validates the effectiveness of the proposed algorithm within a simulated platform environment. Finally, the fifth section offers a summary of the related work.

## II. Trajectory initialization and VOSC obstacle avoidance

### A. Trajectory initialization

In the initial stage of path planning for unmanned aerial vehicles (UAVs), the trajectory is modeled as a smooth and continuous spatial curve that adheres to the UAV’s dynamic constraints [22–24]. When the UAV is modeled as a point mass, this trajectory can be effectively represented using spline interpolation techniques, which offer both computational efficiency and trajectory smoothness. Among these, the B-spline curve has proven to be particularly suitable due to its desirable mathematical properties, such as local support, convex hull containment, and smoothness continuity, which collectively contribute to safe and dynamically feasible path generation [25–27].

The general form of a B-spline curve of order k is expressed as:


$$P\left(S\right)=\sum_{i=0}^{m}P_{i}N_{i,k}\left(s\right)$$ (1)

where $P_{i}$ denotes the ith control point, $N_{i,k}\left(s\right)$ is the B-spline basis function of order k, and s is the normalized curve parameter, defined on the knot vector $\left\{t_{0},t_{1},\cdots t_{m+k}\right\}$.

To simplify the initialization of the control point set $\left\{P_{i}\right\}$, a polynomial fitting-based strategy is adopted. This method allows for rapid determination of control points based on initial and terminal spatial constraints. The number m of control points is determined by:


$$m=\left[\frac{L}{d}\right]$$ (2)

where L is the maximum spatial singular value or Euclidean distance between the start and end points of the desired trajectory, and d is the desired spacing between adjacent control points.

For the basis functions $N_{i,k}\left(s\right)$, a cubic B-spline (k=3) is selected due to its continuous second derivative, which aligns well with the smooth acceleration constraints of UAV motion. The basis functions are recursively defined using the Cox-de Boor recursion formula:


$$\begin{array}{c} N_{i,1}\left(s\right)=\left\{ \begin{array}{c} 1\;if\;t_{i}<s<t_{i}+1\\ 0\;otherwise \end{array}\right.\\ N_{i,k}\left(s\right)=\frac{s-t_{i}}{t_{i+k-1}-t_{i}}N_{i,k-1}\left(s\right)+\frac{t_{i+k}}{t_{i+k}-t_{i+1}}N_{i+1,k-1}\left(s\right) \end{array}$$ (3)

In the implementation, a uniform knot vector is adopted, ensuring equal time allocation among segments. Given the convex hull property of B-spline curves, each trajectory segment remains bounded within the convex envelope formed by its control points. This property ensures safe spatial constraints and simplifies collision avoidance, especially in cluttered or dynamic environments.

Moreover, due to the derivative-preserving nature of B-splines, the first and second derivatives of the trajectory curve also follow B-spline formulations of lower orders. Consequently, the velocity, acceleration, and curvature of the UAV’s trajectory can be efficiently obtained through the same analytical framework. The sharpness or curvature k(s) of the trajectory can be calculated as:


$$k(s)=\frac{\left\|V\left(s\right)\times A(s)\right\|}{{\left\|V\left(s\right)\right\|}^{3}}$$ (4)

The full UAV trajectory is formed by sequentially connecting each segment computed using the control points $P_{i},P_{i+1},P_{i+2},P_{i+3}$, ensuring $C^{2}$ continuity across segment boundaries.

### B. Create 3D velocity collision cone

When the UAV completes trajectory initialization and follows the initial trajectory, it is necessary to predict the trajectory based on the motion states of both the UAV and the dynamic obstacle to ensure effective obstacle avoidance. The classical method for dynamic obstacle avoidance utilizes a Velocity Obstacle Cone to adjust the UAV’s velocity, thereby preventing collisions [28,29].

Assuming that the UAV is situated in three-dimensional space, its velocity is v(t) at time t. At this moment, its motion state can be described as follows: $P_{u}\left(t\right)=\left\{Q_{u}(t),v_{u}(t)\right\}$. where $Q_{u}(t)$ represents the coordinates of the UAV u, and $v_{u}(t)$ denotes the velocity of the UAV. In contrast, the motion state of the obstacle is defined as follows: $P_{o}\left(t\right)=\left\{Q_{o}(t),v_{o}(t)\right\}$. Where $Q_{o}(t)$ indicates the coordinates of the obstacle and $v_{o}(t)$ signifies the velocity of the obstacle.

**Fig 2:**
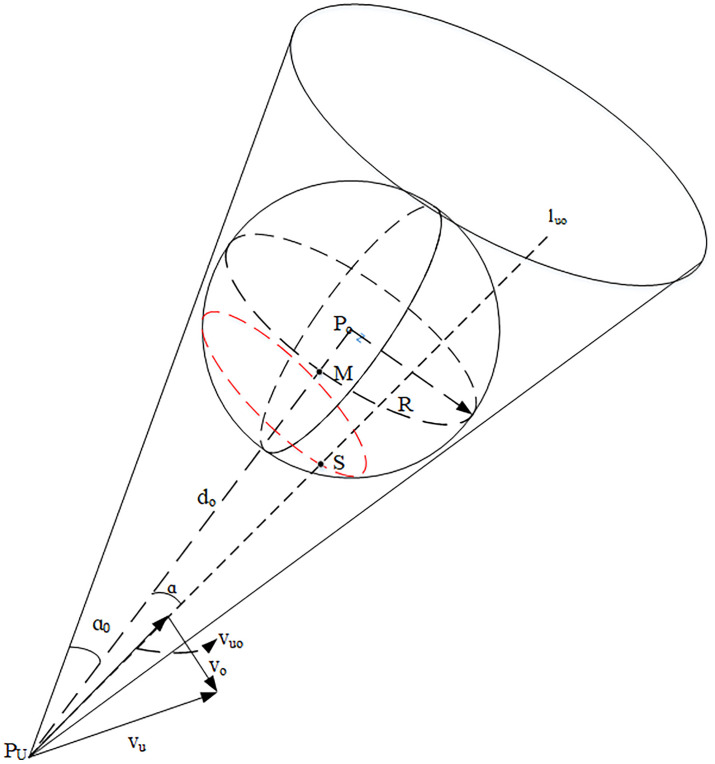
Schematic diagram of 3D velocity collision cone.

The velocity space collision cone consists of the tangent line from the UAV position to the obstacle sphere, and its structure is shown in Fig 2. $Q_{u}$and $Q_{o}$ are the positions of the UAV and the obstacle at the moment t, while the radius of the obstacle sphere *R* is the radius of the expanded obstacle, theR=Kr(k≥1).

The relative speed of the UAV and the obstacle can be expressed as:


$$v_{uo}=v_{u}-v_{o}$$ (5)

Suppose the relative velocity is maintained as constant. In that case, the condition for determining whether the obstacle collides with the UAV is that an intersection exists between the straight line $l_{uo}$ representing the relative velocity and the expanded obstacle sphere O. The ensemble of relative velocities that satisfy the collision is called the 3D relative velocity collision cone (RCC).

The steps to construct the collision cone are outlined as follows:

Step 1: Establish the RCC based on the obstacle avoidance detection distance of the UAV and the radius after the obstacle has been expanded. Physically, it represents the angular range of relative velocities that would inevitably lead to a collision with the obstacle. A larger cone angle implies that the obstacle poses a broader threat region in the velocity space, reducing the UAV’s maneuvering options. The cone angle of the RCC is defined as follows:


$$a_{o}=\arctan\frac{R}{d_{o}}$$ (6)

To compensate for measurement noise and small latency in the relative state, we inflate the obstacle radius used by the relative-velocity cone:


$$\begin{array}{c} R\leftarrow R+\Delta \varepsilon \\ \Delta \varepsilon =z_{1-\delta }\sqrt{n^{T}\sum n} \end{array}$$ (7)

where ∑ is the covariance of the relative position estimate projected onto the contact normal n. $z_{1-\delta }$ denotes the quantile of the standard normal distribution and δ is the allowable risk level. This inflation shrinks the feasible velocity set and yields a conservative, noise-robust collision cone without changing the subsequent derivations.

Step 2: Calculate the angle between the line connecting the obstacle’s center of mass, the UAV’s position, and the relative velocity. This can be expressed mathematically as:


$$\cos a=\cos\left\langle v_{uo},P_{u}P_{o}\right\rangle$$ (8)

where, $\left\|P_{u}P_{o}\right\|=\sqrt{{\left(x_{u}-x_{o}\right)}^{2}+{\left(y_{u}-y_{o}\right)}^{2}+{\left(z_{u}-z_{o}\right)}^{2}}$.

Step 3: Determine the occurrence of a collision:

i: No velocity adjustment is required if the relative velocity is not within the velocity collision cone.

ii: If the relative velocities fall within the velocity collision cone, adjustments must be made to avoid collisions.

$a\in [0,\frac{\pi }{\text{2}})$, if $a\geq \frac{\pi }{\text{2}}$, an effective velocity collision cone cannot be developed, a viable solution remains unattainable.

Furthermore, when the UAV encounters multiple obstacles, it must satisfy various velocity constraints, i.e.,


$$a_{i}>a_{o_{i}},\forall i\in \left\{1,2,\cdots ,n\right\}$$ (9)

### C. Establishment of velocity obstacle spherical cap

**Fig 3:**
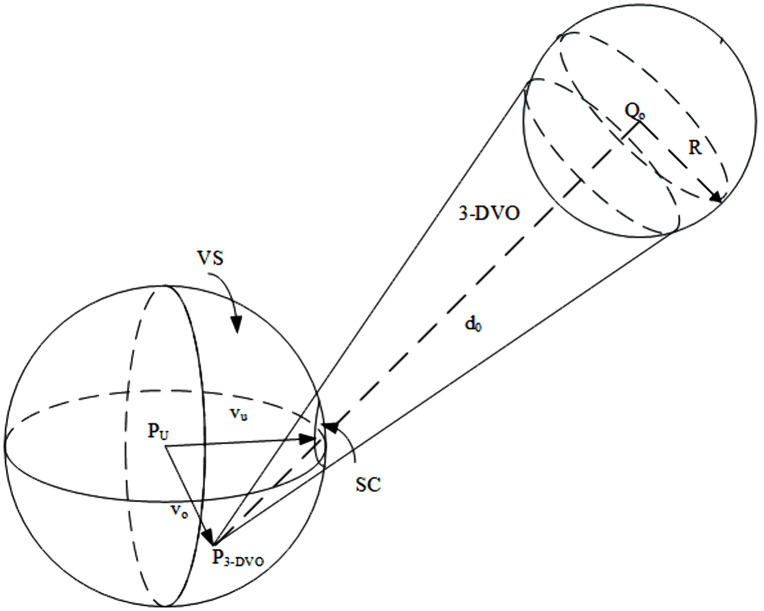
Schematic diagram of UAV’s VOSC.

Obstacle avoidance is achieved by dynamically adjusting the UAV’s velocity vector based on its relative velocity with respect to nearby obstacles, ensuring collision-free motion while maintaining trajectory continuity. Fig 3 schematically illustrates the concept of the UAV’s VOSC. As with the RCC, we inflate the VOSC radius in the same manner to compensate for measurement noise and small sensing latency.

**Fig 4:**
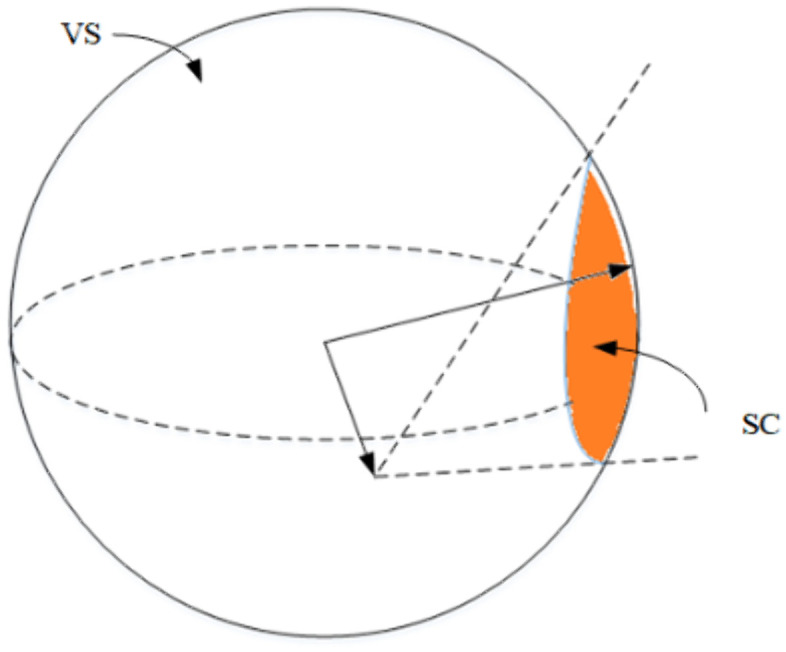
Spherical Crown enlarged image.

Fig 4 shows the spherical crown (SC), representing the boundary region in velocity space where the UAV’s feasible velocities intersect with the dynamic obstacle’s velocity obstacle. Intuitively, this crown-shaped segment defines the critical set of velocities that would lead to a collision, and thus forms the effective risk boundary for trajectory replanning.

**Fig 5:**
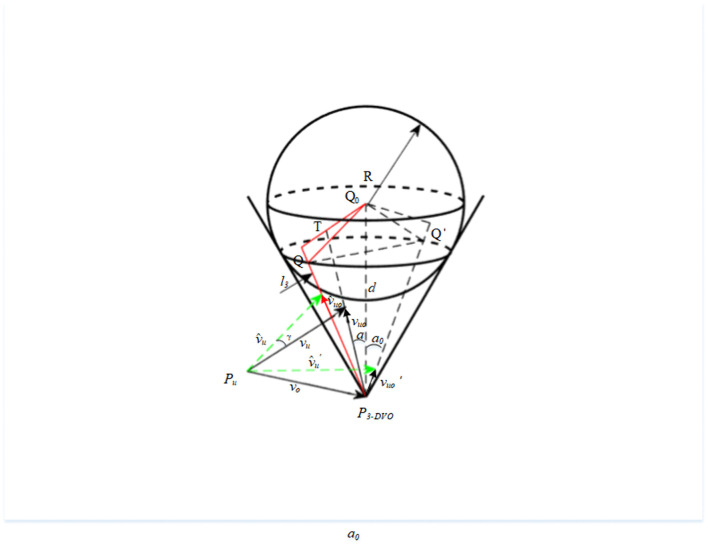
Schematic diagram of the minimum and maximum turning angles of $v_{u}$.

The key to solving the VOSC obstacle avoidance problem lies in determining the critical point of collision, which allows for calculating the minimum deflection angle of the UAV. By minimizing the deflection angle, the UAV preserves trajectory smoothness and avoids unnecessary energy consumption, ensuring that the avoidance maneuver is both dynamically feasible and efficient. In other words, Eq. (10) embodies the principle of minimum disturbance, where the UAV deviates as little as possible from its intended path while still guaranteeing safety. This minimum deflection angle can be mathematically expressed as:


$$\delta =a_{o}-a$$ (10)

where, $a_{o}$ is the half-cone apex angle of the collision cone $P_{3-DVO}$, and a is the angle between the relative velocity and the axis of the collision cone, as shown in Fig 5.

$P_{3-DVO}$ is the cone point of the collision cone and $l_{1},l_{2}$ are the two generatrix of the collision cone. To ensure that the UAV can successfully avoid the obstacle, $l_{3}$ in the plane $P_{3-DVO}Q_{o}T$ and Q is $l_{3}$ the tangent point of the extension line and the obstacle.

A point $P\left(x_{p},y_{p},z_{p}\right)$ on the generatrix $l_{3}$ is selected such that the vector is a unit vector. We can derive further insights from the geometric relationships illustrated in Fig 5.


$$\cos\delta =\frac{v_{uo}\cdot P_{3-DVO}P}{\left\|v_{uo}\right\|\left\|P_{3-DVO}P\right\|}$$ (11)

$$\cos a_{o}=\frac{P_{3-DVO}P\cdot P_{3-DVO}Q_{o}}{\left\|P_{3-DVO}P\right\|\left\|P_{3-DVO}Q_{o}\right\|}$$ (12)

According to the result of the coordinates P, the unit vector q can be expressed as:


$$q=\left(x_{p}-x_{3-DVO},y_{p}-y_{3-DVO},z_{p}-z_{3-DVO}\right)$$ (13)

Therefore, we expect that the relative velocity ${\hat{v}}_{uo}$ can be expressed as:


$${\hat{v}}_{uo}=\lambda q$$ (14)

where, λ represents the size of the expected relative velocity ${\hat{v}}_{uo}$.

Assuming that the velocity of the obstacle $v_{o}$remains constant during the period T, the angle between the velocity of the obstacle $v_{o}$ and the expected relative velocity vector ${\hat{v}}_{uo}$ can be determined as follows:


$$\cos\left\langle v_{o},{\hat{v}}_{uo}\right\rangle =\frac{v_{o}\left(\lambda q\right)}{\left\|v_{o}\right\|\left\|{\hat{v}}_{uo}\right\|}=\frac{v_{o}\cdot q}{\left\|v_{o}\right\|}$$ (15)

Consequently, the vector ${\hat{v}}_{uo}$ can be determined as follows:


$$\begin{array}{c} {\hat{v}}_{uo}=\left\|v_{o}\right\|\cos\left(\pi -\left\langle v_{o},{\hat{v}}_{uo}\right\rangle \right)\\ +\sqrt{{\left\|v_{o}\right\|}^{2}\cos^{2}\left\langle v_{o},{\hat{v}}_{uo}\right\rangle -\left({\left\|v_{o}\right\|}^{2}-{\left\|v_{u}\right\|}^{2}\right)} \end{array}$$ (16)

Thus, the expected velocity expectation value can be expressed as follows:


$${\hat{v}}_{u}=\left[\begin{array}{c} \left\|v_{o}\right\|\cos\theta_{o}\cos\psi_{o}+\left\|{\hat{v}}_{uo}\right\|\left(x_{p}-x_{3-DVO}\right)\\ \left\|v_{o}\right\|\cos\theta_{o}\sin\psi_{o}+\left\|{\hat{v}}_{uo}\right\|\left(y_{p}-y_{3-DVO}\right)\\ \left\|v_{o}\right\|\sin\theta_{o}+\left\|{\hat{v}}_{uo}\right\|\left(z_{p}-z_{3-DVO}\right) \end{array}\right]$$ (17)

Therefore, the expected direction angle of the UAV’s velocity is:


$$\cos{\hat{\psi }}_{u}=\frac{{\hat{v}}_{ux}}{\sqrt{{\hat{v}}_{ux}^{2}+{\hat{v}}_{uy}^{2}}}$$ (18)

$$\sin{\hat{\theta }}_{u}=\frac{{\hat{v}}_{uz}}{\sqrt{{\hat{v}}_{ux}^{2}+{\hat{v}}_{uy}^{2}+{\hat{v}}_{uz}^{2}}}$$ (19)

The minimum deflection angle (Δψ,Δθ) of the UAV’s velocity is:


$$\begin{array}{c} \Delta \psi ={\hat{\psi }}_{u}-\psi_{u}\\ \Delta \theta ={\hat{\theta }}_{u}-\theta_{u} \end{array}$$ (20)

where, $\psi_{u}$ and $\theta_{u}$ are the direction angle of the original velocity of the UAV. The relationship between the expected velocity and the original velocity of the UAV can be expressed as follows:


$${\hat{v}}_{u}=𝐀v_{u}$$ (21)

$$𝐀=\left[\begin{array}{cc} {}*20c\cos\Delta \theta & \sin\Delta \theta \\ -\sin\Delta \theta & \cos\Delta \theta \end{array}\right]\left[\begin{array}{cc} {}*20c\cos\Delta \psi & \sin\Delta \psi \\ -\sin\Delta \psi & \cos\Delta \psi \end{array}\right]$$ (22)

**Fig 6:**
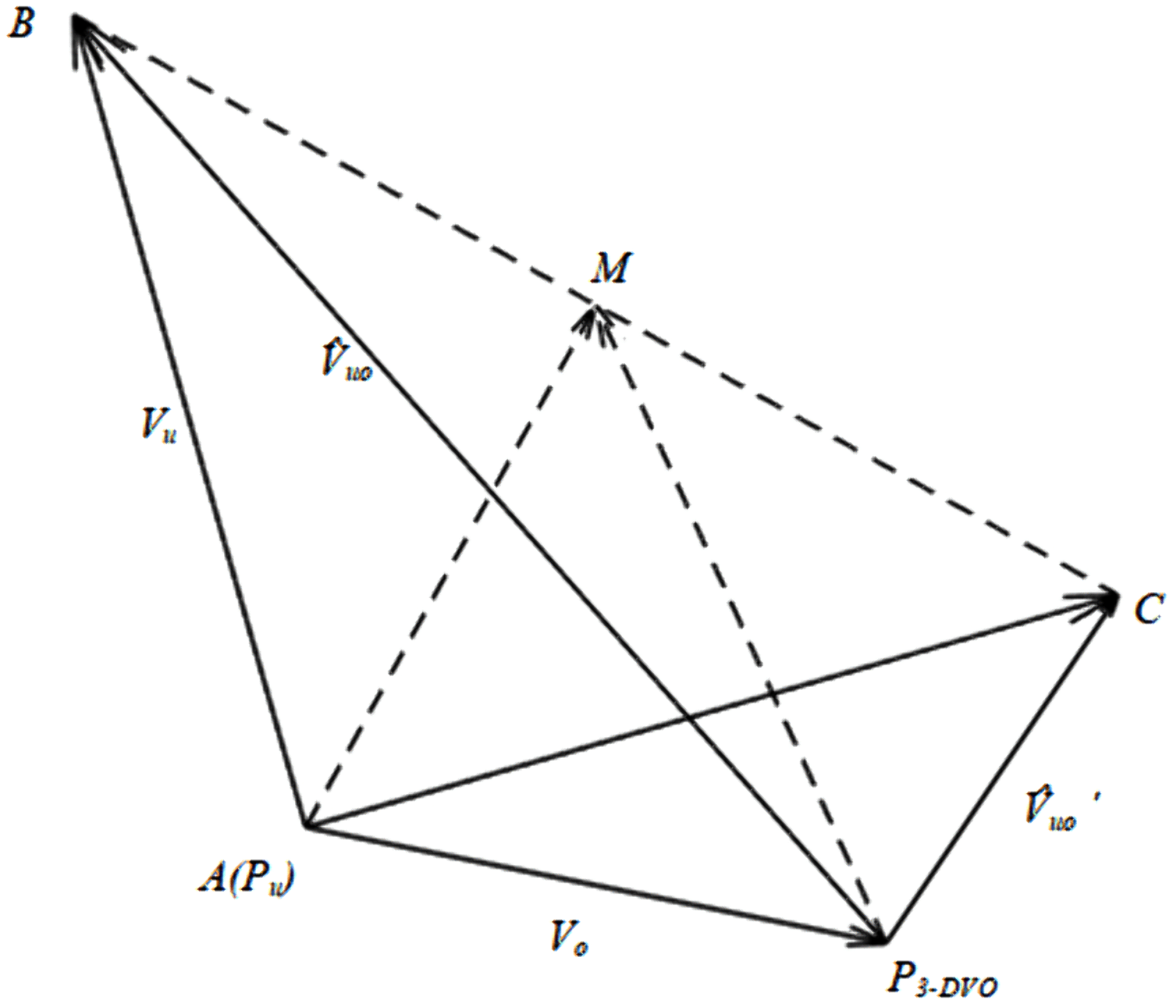
VOSC vector diagram.

Based on the UAV’s expected speed, the parameters of its VOSC can be determined. Fig 6 illustrates the obstacle avoidance space vector diagram corresponding to the UAV’s VOSC. Point *M* represents the midpoint of the line segment *BC*, which can be derived from the expected velocity vectors ${\hat{v}}_{uo}$ and ${\hat{v}}_{uo}^{\prime }$.

According to the geometric relations of space $AM=P_{3-DVO}M+AP_{3-DVO}$, it is known that:


$$AM=v_{o}+\frac{\text{1}}{\text{2}}\left({\hat{v}}_{uo}+{\hat{v}}_{uo}^{\prime }\right)=\left(x_{AM},y_{AM},z_{AM}\right)$$ (23)

**Fig 7:**
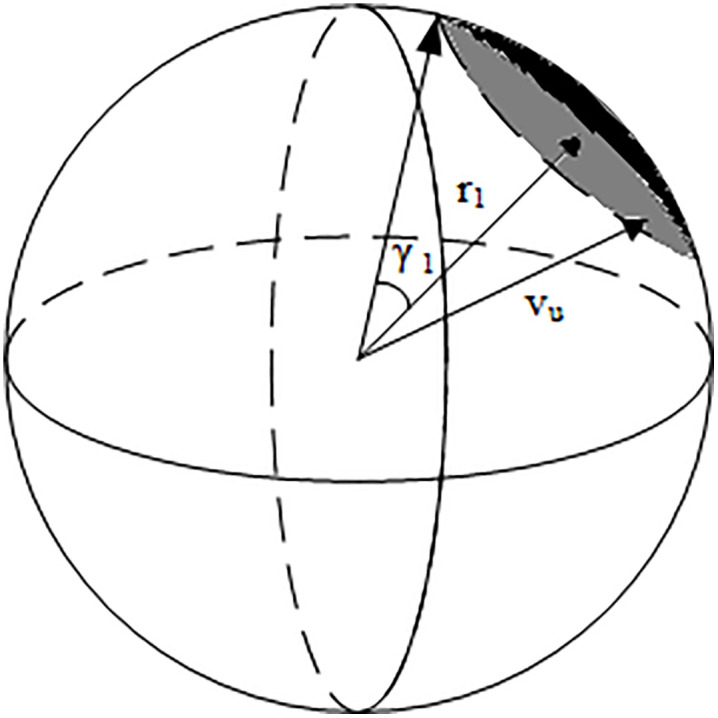
Schematic diagram of VOSC for a single obstacle.

The VOSC spatial parameters $\left(r,\psi_{r},\theta_{r},\gamma \right)$of the obstacle O can be expressed as follows:


$$\cos\psi_{r}=\frac{x_{AM}}{\sqrt{x_{AM}^{2}+y_{AM}^{2}}}$$ (24)

$$\sin\theta_{r}=\frac{z_{AM}}{\sqrt{x_{AM}^{2}+y_{AM}^{2}+z_{AM}^{2}}}$$ (25)

$$\sin\gamma =\frac{\left\|BC\right\|}{2\left\|v_{u}\right\|}$$ (26)

where, *r* is the size of SC vectors $P_{u}M$, $\psi_{r}$, $\theta_{r}$ is the direction angle of the vector *AM*, and the four parameters quantify the impact of the dynamic obstacle O on the UAV, the structure of which is shown in Fig 7.

## III. Dynamic obstacle avoidance direction solving and trajectory replanning optimization

### A. Obstacle avoidance direction solving with VOSC constraints

In a single obstacle scenario, obstacle avoidance represents the simplest and most common situation. In this context, it is necessary to adjust the velocity direction of the UAV outside the three-dimensional (3D) velocity obstacle space. To achieve minimum deflection angle obstacle avoidance, the relative velocity ${\hat{v}}_{uo}$ direction of the UAV and the obstacle must align with the generatrix $l_{3}$ direction of the 3D velocity obstacle space collision cone. Based on the parameter solutions in the velocity obstacle space, the desired velocity ${\hat{v}}_{u}$ of the UAV can be further determined.

**Fig 8:**
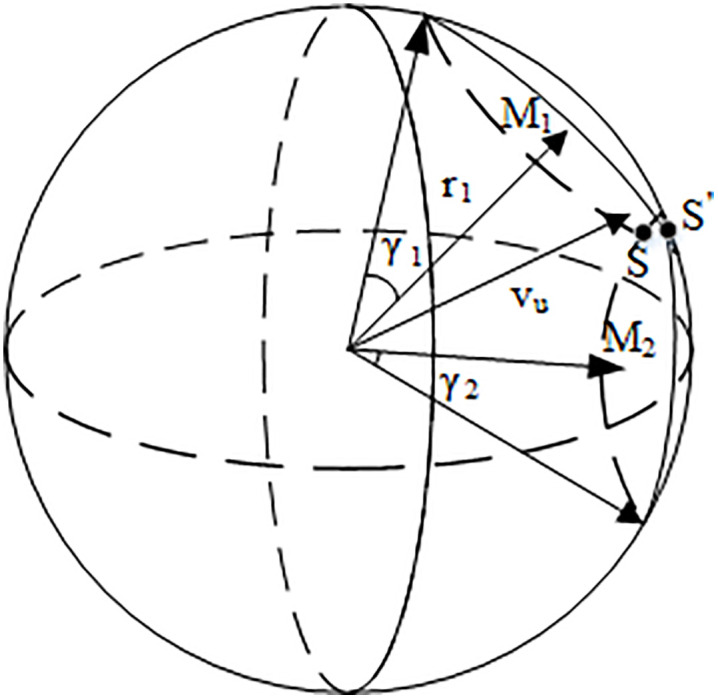
Schematic diagram of VOSC with multiple obstacles.

In multi-obstacle scenarios, it is essential first to ascertain whether an intersection exists within the Velocity Obstacle Set VOSC space of multiple obstacles. If no intersection is identified, the obstacle avoidance strategy applicable to a single obstacle can be employed. Conversely, if an intersection does occur, it becomes imperative to select the direction of velocity deflection judiciously. For illustration, consider the VOSC space of two threatening obstacles, as depicted in Fig 8. The parameters defining the VOSC space for these two threatening obstacles are denoted as $G_{O_{1}}\left(r_{1},\psi_{1},\theta_{1},\gamma_{1}\right)$and$G_{O_{2}}\left(r_{2},\psi_{2},\theta_{2},\gamma_{2}\right)$. Where $r_{1}$ and $r_{2}$respectively represent the size of the vector $P_{u}M_{1}$ and $P_{u}M_{2}$.$\left(\psi_{1},\theta_{1}\right)$ and $\left(\psi_{2},\theta_{2}\right)$ respectively represent the direction angle of the two vectors, $\gamma_{1}$and $\gamma_{2}$ respectively represent the size of two three-dimensional space VOSC.

Fig 8 illustrates that when confronted with two threatening obstacles, the direction of the UAV’s critical state velocity for obstacle avoidance should be the intersection of the two VOSC great circles intersection point S and $S^{\prime }$. Utilizing the geometrical relationships, $P_{u}M_{1}⊥M_{1}S$, $P_{u}M_{2}⊥M_{2}S$ the coordinates of this intersection point in the critical state of obstacle avoidance can be determined. Subsequently, the expected UAV velocity corresponding to the minimum deflection angle can be calculated, yielding the following expression.


$${\hat{v}}_{u}=\left\|P_{u}S\right\|$$ (27)

When confronted with three or more threatening obstacles, it is essential to analyze further whether the intersection point of the VOSC great circle can satisfy the condition of simultaneously avoiding multiple obstacles. In such cases, the boundary point of the VOSC concatenation can be utilized to determine the direction of the UAV’s obstacle avoidance velocity vector, with any point *K* on the boundary representing a critical point in the obstacle avoidance state. Based on the geometric relationships involved, the expected speed ${\hat{v}}_{u}=\left\|P_{u}K\right\|$ of the UAV in this critical state can be calculated. Combined with previously established methods, this calculation facilitates the completion of the VOSC parameter solution.

### B. Safe trajectory replanning and velocity adjustment

If the velocity $v_{o}$ of the obstacle remains constant during the interval T, the expression for the position of the obstacle after the avoidance time T can be expressed as follows:


$$P_{o}^{\prime }=\left[\begin{array}{c} x_{o}^{\prime }\\ y_{o}^{\prime }\\ z_{o}^{\prime } \end{array}\right]=\left[\begin{array}{c} x_{o}+(v_{o}\cos\theta_{o}\cos\psi_{o})T\\ y_{o}+(v_{o}\cos\theta_{o}\sin\psi_{o})T\\ z_{o}+\left(v_{o}\sin\theta_{o}\right)T \end{array}\right]$$ (28)

To ensure the safety of UAV flight, the replanning insertion point is positioned in the opposite direction of the obstacle’s velocity. If the radius of obstacle expansion is multiplied by the expansion factor *k*, then the coordinates of the obstacle avoidance replanning insertion point are:


$$Q_{avo}=\left[\begin{array}{c} x_{avo}\\ y_{avo}\\ z_{avo} \end{array}\right]=\left[\begin{array}{c} x_{o}^{\prime }+R^{\prime }\cos\theta_{o}\cos\psi_{o}\\ y_{o}^{\prime }+R^{\prime }\cos\theta_{o}sim\psi_{o}\\ z_{o}^{\prime }+R^{\prime }\sin\theta_{o} \end{array}\right]$$ (29)

where, $R^{\prime }=kR$, and k>1.

In a multi-obstacle situation, if there are m obstacles, the obstacle avoidance guidance time for each obstacle is $T_{i}(i=1,2,\cdots ,m)$, then the shorter collision time is selected to be the UAV obstacle avoidance guidance time.


$$T=\min(T_{1},T_{2},\cdots ,T_{m})$$ (30)

where, $T_{i}=\frac{\left\|d_{o}-R_{i}\right\|}{\left\|v_{o}\right\|}$, $d_{o}$ is the distance between the estimated collision point and the current position of the obstacle.

The desired velocity of the UAV at the minimum deflection angle can be calculated by considering the effects of dynamic obstacles on the UAV, as determined in Part II A, over the time interval *T*. The desired velocity of the UAV at the minimum deflection angle can be calculated by considering the effects of dynamic obstacles on the UAV. Based on the difference between the UAV’s current velocity $v_{u}$ and the desired velocity ${\hat{v}}_{u}$, combined with the maximum acceleration $a_{\max}$ of the UAV, the time *t* required for the velocity change is solved. After that, the velocity change curve of the UAV is fitted according to *t*he method in the trajectory initialization in the previous section A, and the trajectory of the UAV’s velocity change is generated based on this curve. *t* is expressed as:


$$t=\max(\frac{{\hat{v}}_{ux}-v_{ux}}{a_{\max}},\frac{{\hat{v}}_{uy}-v_{uy}}{a_{\max}},\frac{{\hat{v}}_{uz}-v_{uz}}{a_{\max}})$$ (31)

where, *t* is a small fragment of time *T*, whose value is limited by the UAV’s capabilities, this velocity fitting method ensures not only the smoothness of the UAV trajectory but also that the trajectory consistently adheres to the UAV’s performance constraints.

The VOSC-based collision-avoidance procedure is summarized in Algorithm 1.

Algorithm 1: Online VOSC Replanner

Input: UAV state: position $P_{u}$, velocity $v_{u}$

  Obstacles state: $O={\left\{\left(P_{i},v_{i},R\right)\right\}}_{i=1\ldots M}$

  Initial trajectory: P(S),

Output: New trajectory: $P_{new}\left(S\right)$ and $v_{cmd}$

Insert $O_{i}$ into Potential-threat set:

while Threats not empty do

 Establish VOSC model:

  $paramerer_{i}$ ← Build_VOSC(Threats, $P_{u}$, $v_{u}$)

   if Threats = 1 then

    δ ← MinDeflectionSingle($paramerer_{i}$)

   else

    δ ←MinDeflectionMultiple($paramerer_{i},\cdots paramerer_{j}$)

   end if

${\hat{v}}_{u}$ ← ShapeSpeed(δ,$v_{u}$,$v_{\max}$)

$P_{new}\left(S\right)$=FitLocalB-Spline(P(S))

$v_{cmd}$ ←Timeadjustment ($P_{new}\left(S\right)$)

while end

## IV. Simulation experiments

To verify the effectiveness of the proposed obstacle avoidance algorithm, simulation experiments were conducted using the Robot Operating System and the open-source flight control PX4, with results visualized in the RVIZ software [30]. To focus on validating the core algorithmic performance, the simulations are conducted under idealized conditions. UAV dynamics and environmental disturbances are not considered.

This setup isolates the effects of the proposed planner on trajectory generation and obstacle avoidance.

The simulation of obstacle avoidance in a dynamic environment comprises three main steps:

1. Global Path Planning: The B-spline algorithm is employed for global path planning, ensuring both the smoothness and efficiency of the path while enabling rapid generation of the global trajectory.
2. Path Following and Obstacle Detection: The UAV follows the B-spline planned trajectory and continuously monitors obstacles within a specified range to assess their potential impact on flight.
3. Determining the Obstacle Avoidance Strategy: For obstacles, the 3D velocity obstacle cone algorithm calculates the minimum deflection angle, allowing for adjustments in flight direction to effectively avoid obstacles.

### A. Single dynamic obstacle environments

**Fig 9:**
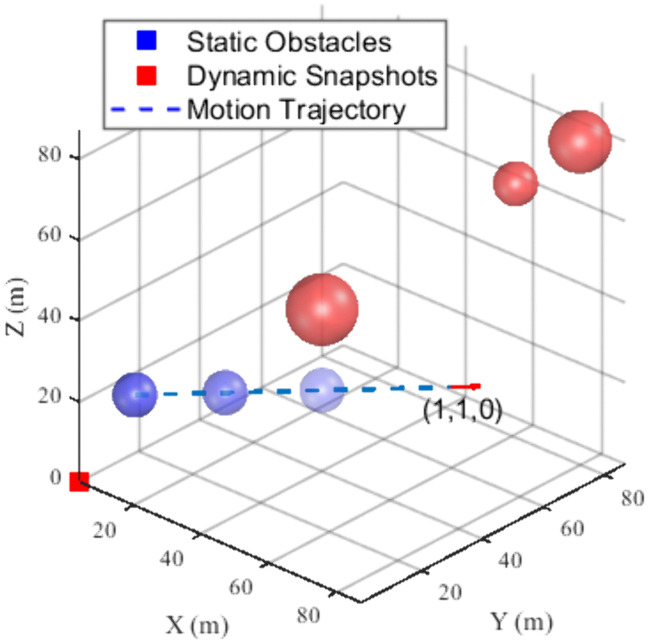
Single dynamic obstacle simulation environment.

During the simulation experiments, the effects of UAV dynamics and environmental factors were not considered to illustrate the performance of the algorithm better. In the experiment conducted within a single dynamic obstacle environment, we configured the UAV to traverse from the starting point (0, 0, 0) to the target point (100, 100, 100). The UAV is characterized by a maximum acceleration of 5 m/s^2^ and a flight speed of 5 m/s along all three axes, and the UAV’s detection range is 15 m. Four spherical obstacles were incorporated into the simulation environment, including a dynamic obstacle with a radius of 5 meters, positioned initially at (10, 10, 20), which moves along the x and y axes at a speed of (1, 1, 0). Furthermore, static obstacles are located at (70, 70, 70), (80, 80, 80), and (40, 40, 40), with respective radii of 5, 7, 7 m. A schematic diagram of the simulation environment is presented in Fig 9. In this fig, the blue sphere denotes the dynamic obstacle, the grey spheres represent the static obstacles, and the direction indicated by the red arrow illustrates the motion path of the dynamic obstacle.

**Fig 10:**
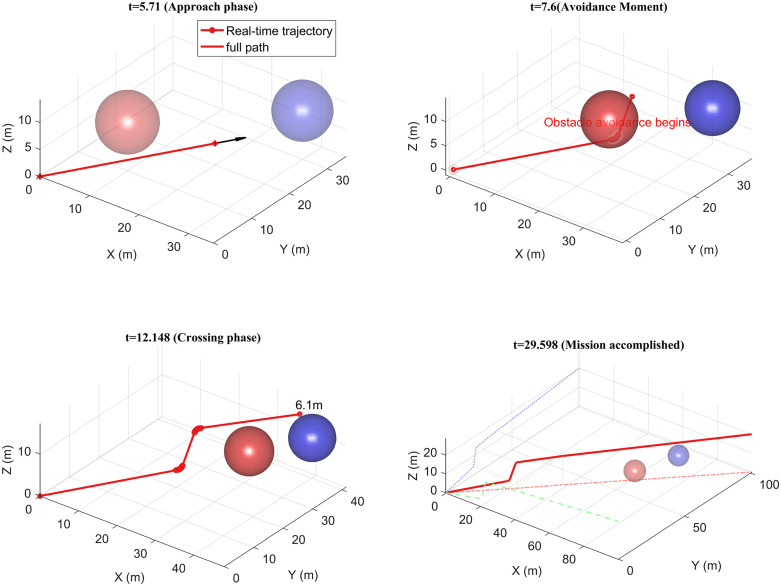
UAV trajectory diagram for simulation experiment 1.

To validate the effectiveness of the proposed algorithm, we conducted comparative experiments with representative path planning methods for dynamic environments, including the Dynamic Window Approach (DWA), the B-spline path planning algorithm, and the hybrid CPU/GPU implementation of the Asynchronous Advantage Actor-Critic–based Collision Avoidance with Deep Reinforcement Learning (GA3C-CADRL) algorithm [30]. The proposed method exhibits the capability to accurately predict obstacle motion and adjust the UAV’s trajectory in real time when potential collisions are detected. This enables the UAV to achieve minimum-distance avoidance of dynamic obstacles while maintaining smooth and dynamically feasible trajectories. The UAV’s motion path under the proposed 3D planning framework is illustrated in Fig 10, and the corresponding quantitative results are summarized in Table 1. During the dynamic obstacle avoidance process, the VOSC parameters are recorded as (7.97, 45°, 55.9°, 23.07°).

**Table 1:** Trajectory related information in Experiment 1.

Algorithm	Length(m)	Time(s)
Proposed	167.07	21.04
DWA	189.50	24.816
B-spline	203.97	26.69
GA3C-CADRL	186.40	24.35

As illustrated in Table 1 and Fig 10, the proposed algorithm reduces path length metrics by approximately 7.09% and 13.68% when compared to the DWA and the B-spline algorithms, respectively, while also achieving superior trajectory smoothness. When compared with the GA3C-CADRL method, the proposed algorithm achieves a further 5.54% reduction in trajectory length and shortens the flight time by about 13.5%, demonstrating both higher efficiency and improved real-time adaptability.

**Fig 11:**
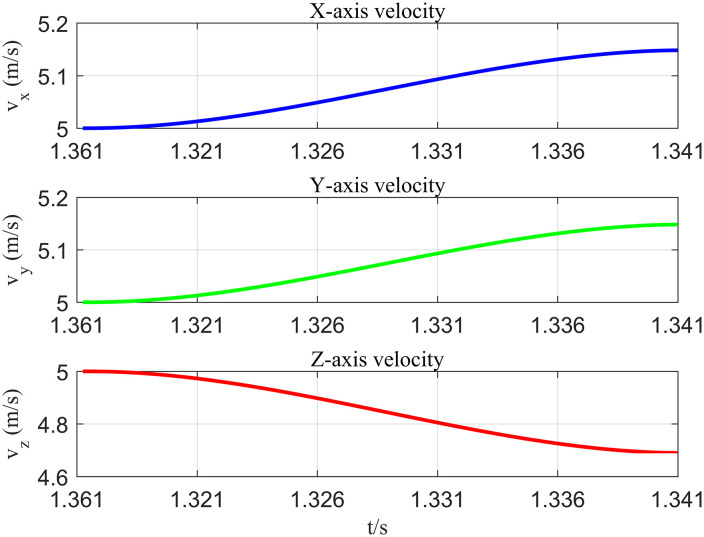
Velocity transformation curve of the UAV when avoiding dynamic obstacles.

When the relative distance between the UAV and the dynamic obstacle reaches the 15m warning threshold, the proposed algorithm completes the smooth adjustment of the 3D velocity vector within 0.051 seconds by integrating the obstacle motion prediction model with the B-spline trajectory optimizer, as illustrated in Fig 11. After the UAV detects the dynamic threat at 1.316 seconds, it forecasts the obstacle’s trajectory for the subsequent 5 seconds by formulating its kinematic differential equation. It constructs an optimal control model under acceleration constraints, ultimately generating new velocity vectors that conform to the UAV’s dynamic characteristics at 1.367 seconds (v_x_ = 5.18 m/s, v_y_ = 5.18 m/s, v_z_ = 4.69 m/s). The entire obstacle avoidance process took a total of 1.812 seconds. Regression analysis indicated that the obstacle avoidance time exhibited a linear positive correlation with the detection distance and an exponentially negative correlation with the relative speed of the obstacle.

### B. Multi-dynamic obstacle environments

**Fig 12:**
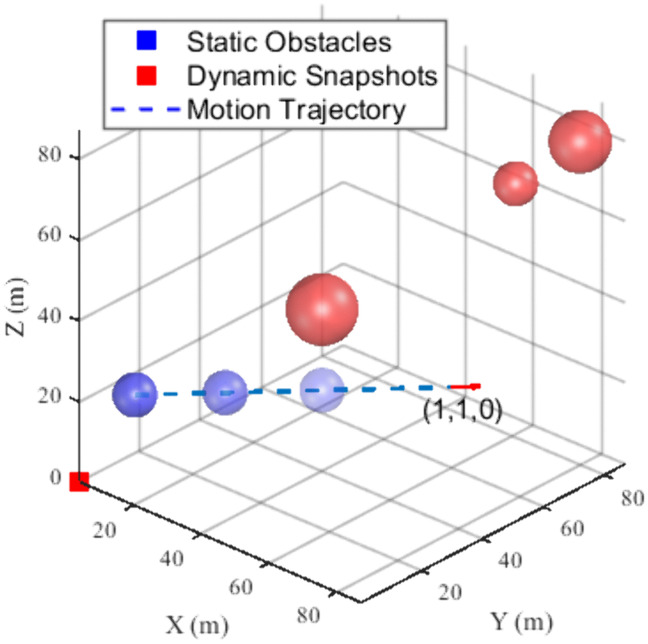
Initial environment for multi-dynamic obstacles.

In the context of a multi-dynamic obstacle scenario, the situation becomes more complex, making it more challenging to ascertain the direction of the minimum deflection angle for the UAV. In this experiment, we introduced two dynamic obstacles, the relevant parameters detailed in Table 2. Fig 12 presents the settings of the simulation environment.

**Table 2:** Parameters related to dynamic obstacles.

Initial condition	$O_{1}$	$O_{2}$
Position coordinate	(18.58,24.29,7.145)	(−19.69,-19.69,11.28)
Velocity	(2,1,0.5)	(5.2,5.2,-0.4)
Swelled radius	5	5
Threat judgment	$a_{1}<a_{o1}$	$a_{2}<a_{o2}$
SC parameter	(2.16,135,61.3,64.4)	(4.89,36.5,16.3, 11.91)

**Fig 13:**
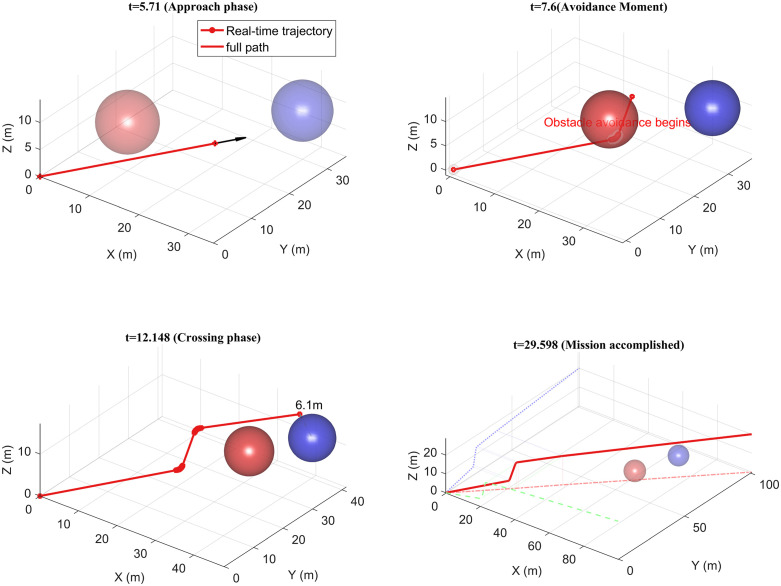
UAV trajectory in a multi-dynamic obstacle environment.

The UAV operates within a three-dimensional space, commencing its flight from the coordinates (0,0,0) and reaching the destination at (100,100,20) with a maximum speed of 10 m/s. The UAV’s detection range is 15 m, which is illustrated in Fig 13, depicting both the UAV’s trajectory and the trajectory of the dynamic obstacle.

The experiment illustrated in Fig 13 validates the efficacy of the UAV dynamic obstacle avoidance trajectory planning algorithm. The experimental process comprises four key phases: (a) the initial approach phase, during which the UAV advances towards the target point along the planned path at a speed of 5 m/s; (b) the dynamic obstacle avoidance phase, wherein the obstacle avoidance mechanism is activated when the distance to the obstacle falls below the detection threshold of 15 m. In this phase, a multi-obstacle avoidance strategy based on the minimum deflection angle criterion generates a speed of (1.442, 1.442, 4.564) m/s, maintained for a duration of 1.89 s; (c) the trajectory replanning phase, where the trajectory update following the initial obstacle avoidance remains influenced by obstacles, prompting the activation of a secondary obstacle avoidance strategy. The speed is adjusted to (3.46, 3.58, 0.368) m/s and sustained for 4.55s; (d) the trajectory convergence phase, culminating in a total flight trajectory length of 149.002 m and a time duration of 29.6 s.

The experimental data demonstrate that the 3D trajectory curves, as projected onto the x, y, and z axes (illustrated by the dotted line), maintain C1 continuity. This effectively prevents abrupt changes in the trajectory. Notably, a quantitative analysis of the velocity vector and the duration of obstacle avoidance in the proximity of obstacles confirms that the method achieves: 1) smooth articulation of multi-stage obstacle avoidance maneuvers; 2) progressive optimization of 3D trajectory curvature; 3) An effective balance between avoidance efficiency and trajectory optimality in complex dynamic environments. Furthermore, the energy consumption associated with obstacle avoidance is reduced, underscoring its practical engineering value.

**Fig 14:**
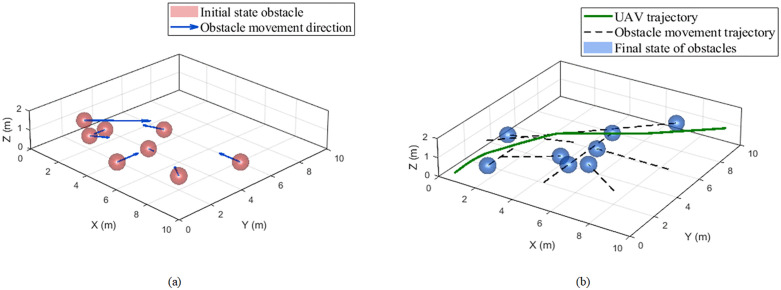
Drone trajectory map under 8 moving obstacles.

To assess feasibility under multiple dynamic obstacles, we consider a 10 × 10 × 3 m workspace with randomized moving–obstacle sets. A micro-UAV departs from (0.5, 0.5, 0.1) and navigates to (9.5, 9.5, 1.0), subject to a maximum speed of 2 m/s, a sensing range of 2 m, a maximum acceleration of 6 m/s2, and a body radius of 0.2 m. Each scenario is repeated 10 times with different random seeds, and we report averages over these trials. Results are presented in the corresponding Fig 14 and in Table 3.

**Table 3:** Performance vs. number of moving obstacles.

Obstacles	Success Rate	Path Length (m)	Flight Time (s)	Jerk Smoothness $\int {\left\|j\right\|}^{2}dt$	$d_{\min}$(m)
2	1	13.10	9.47s	30.75	0.83
4	1	13.21	9.8s	41.56	0.86
6	1	13.32	10.12s	35.51	0.79
8	1	13.54	10.23s	33.93	0..75

Under a stringent geometric setting where all obstacles have a 0.4 m radius, and where obstacle positions and speeds are randomly sampled, the proposed method maintains a 100% success rate across scenarios with 2–8 moving obstacles, with only marginal increases in path length and flight time, consistently favorable jerk-based smoothness, and safety margins well above the 0.6 m contact threshold, demonstrating safety, smoothness, and scalability in dense dynamic environments.

## V. Conclusions and future work

This study proposed a comprehensive set of real-time obstacle avoidance and trajectory planning solutions to the dynamic obstacle avoidance and trajectory optimization problem for UAVs. Establishing a four-parameter quantitative model based on VOSC effectively assesses the spatial threat level posed by dynamic obstacles to UAVs. Building on this assessment, a fast solution algorithm for minimizing the deflection angle is introduced, significantly enhancing computational efficiency. Additionally, we present a dynamic adjustment mechanism for the 3D velocity vector, which equips UAVs with a high degree of freedom in motion planning within complex obstacle environments. The integration of the adaptive B-spline curve fitting algorithm significantly reduces the trajectory curvature change rate, thereby enhancing both the smoothness and energy efficiency of the flight trajectory. The proposed model for determining insertion points during trajectory replanning effectively addresses the challenges associated with UAV path replanning, ensuring that the UAV can swiftly complete its flight mission while minimizing range waste. Simulation results demonstrate that this study enables UAVs to avoid obstacles in dynamic environments with dense obstacles effectively.

As future work, we will deploy the planner on micro-UAV onboard hardware and perform real-flight experiments. We will further study fast flight computation in indoor environments with onboard sensors, and systematically evaluate how external factors influence the algorithm’s performance.

## Supporting information

S1 File The UAV trajectory data corresponding to Fig 14 in the manuscript. (CSV)

## References

[1] McGuire KN, De Wagter C, Tuyls K, Kappen HJ, de Croon GCHE. Minimal navigation solution for a swarm of tiny flying robots to explore an unknown environment. Sci Robot. 2019;4(35):eaaw9710. doi: 10.1126/scirobotics.aaw9710 33137730

[2] Li Y, Li A, Zhang Z, Song G. Fault-tolerant control of nonlinear cluster system for fixed-wing UAV piston engine faults based on hierarchical architecture. Aerospace Sci Technol. 2025;157:109804. doi: 10.1016/j.ast.2024.109804

[3] Hönig W, et al. Trajectory planning for quadrotor swarms. IEEE Trans Robot. 2018;34:856–69.

[4] Quan L, Han L, Zhou B, Shen S, Gao F. Survey of UAV motion planning. IET Cyber-Syst Robot. 2020;2(1):14–21. doi: 10.1049/iet-csr.2020.0004

[5] Egerstedt M, Xiaoming Hu. Formation constrained multi-agent control. IEEE Trans Robot Automat. 2001;17(6):947–51. doi: 10.1109/70.976029

[6] Li Q, et al. Collaborative trajectory planning for hypersonic vehicles considering angle constraints. Springer Nature Singapore. Vol. 15. 2025. pp. 206–16.

[7] Junwei Z, Jianjun Z. Path Planning of Multi-UAVs Concealment Attack Based on New A* Method. Sixth International Conference on Intelligent Human-Machine Systems and Cybernetics. Vol. 2. IEEE; 2014. pp. 401–4. doi: 10.1109/ihmsc.2014.198

[8] Jianya. An efficient implementation of shortest path algorithm based on dijkstra algorithm. J Wuhan Tech Univ Survey Mapp. 1999;3(004):1–10.

[9] Xu G, et al. An improved A* algorithm based on bidirectional search. Springer Nature Singapore. 2024. pp. 314–22.

[10] Zhou B, et al. Robust real-time UAV replanning using guided gradient-based optimization and topological paths. In: ICRA. Paris: 2020. pp. 1208–14.

[11] Yang X. Development of Obstacle Avoidance for Autonomous Vehicles and an Optimization Scheme for the Artificial Potential Field Method. 2021 2nd International Conference on Computing and Data Science (CDS). 2021. pp. 12–8. doi: 10.1109/cds52072.2021.00010

[12] Lingsong D et al. Research on Trajectory Tracking and Point Control Strategies for Multi-rotor UAVs. Springer Nature Singapore. 2025. pp. 141–52.

[13] Khial N, Mhaisen N, Mabrok M, Mohamed A. An online learning framework for UAV search mission in adversarial environments. Expert Syst Appl. 2025;267:126136. doi: 10.1016/j.eswa.2024.126136

[14] Zhang Z, Liu X, Feng B. Research on obstacle avoidance path planning of UAV in complex environments based on improved Bézier curve. Sci Rep. 2023;13(1):16453. doi: 10.1038/s41598-023-43783-7 37777586 PMC10542762

[15] Senko V, et al. Real-time trajectory replanning for MAVs using uniform b-splines and a 3D circular buffer. In: Proceedings of the 2017 IEEE/RSJ International Conference on Intelligent Robots and Systems (IROS). Canada; 2017. pp. 24–8.

[16] Guo C, Huang L, Tian K. Combinatorial optimization for UAV swarm path planning and task assignment in multi-obstacle battlefield environment. Appl Soft Comput. 2025;171:112773. doi: 10.1016/j.asoc.2025.112773

[17] Lee HD, Kim C, Hur SW, Lee S. A Study on the Flight Path Generation Using Polynomials and Tracking Control. J Inst Control Robot Syst. 2018;24(11):1059–68. doi: 10.5302/j.icros.2018.18.0102

[18] Mellinger D, Kumar V. Minimum snap trajectory generation and control for quadrotors. In: 2011 IEEE International Conference on Robotics and Automation. 2011. pp. 2520–5. doi: 10.1109/icra.2011.5980409

[19] Snape J, Berg JVD, Guy SJ, Manocha D. The hybrid reciprocal velocity obstacle. IEEE Trans Robot. 2011;27(4):696–706. doi: 10.1109/tro.2011.2120810

[20] Han R, Chen S, Wang S, Zhang Z, Gao R, Hao Q, et al. Reinforcement learned distributed multi-robot navigation with reciprocal velocity obstacle shaped rewards. IEEE Robot Autom Lett. 2022;7(3):5896–903. doi: 10.1109/lra.2022.3161699

[21] Alejo D, Cobano JA, Heredia G, Ollero A. Optimal Reciprocal Collision Avoidance with mobile and static obstacles for multi-UAV systems. In: 2014 International Conference on Unmanned Aircraft Systems (ICUAS). Oriando, FL, USA: 2014. pp. 1259–66. doi: 10.1109/icuas.2014.6842383

[22] Huang H, Zhou H, Zheng M, Xu C, Zhang X, Xiong W. Cooperative Collision Avoidance Method for Multi-UAV Based on Kalman Filter and Model Predictive Control. In: 2019 IEEE International Conference on Unmanned Systems and Artificial Intelligence (ICUSAI). IEEE; 2019. pp. 1–7. doi: 10.1109/icusai47366.2019.9124863

[23] Lihaoqi Z, et al. Enhancing the Heuristic Function of Improved A* Algorithm for UAV Robotic Arm Path Planning Using Dynamic Pigeon-Inspired Optimization. Springer Nature Singapore; 2025. pp. 152–61.

[24] Van den Berg J, Lin M, Manocha D. Reciprocal velocity obstacles for real-time multi-agent navigation. In: 2008 IEEE International Conference on Robotics and Automation. IEEE; 2008. pp. 1928–35.

[25] Zhao S, Zheng T, Wang C, Yang Z, Xu T, Zhu Y, et al. Sigmoid angle-arc curves: enhancing robot time-optimal path parameterization for high-order smooth motion. Robot Comput-Integr Manufact. 2025;92:102884. doi: 10.1016/j.rcim.2024.102884

[26] Ding W, Gao W, Wang K, Shen S. An efficient B-spline-based kinodynamic replanning framework for quadrotors. IEEE Trans Robot. 2019;35(6):1287–306. doi: 10.1109/tro.2019.2926390

[27] Zhou X, Wang Z, Ye H, Xu C, Gao F. EGO-planner: An ESDF-free gradient-based local planner for quadrotors. IEEE Robot Autom Lett. 2021;6(2):478–85. doi: 10.1109/lra.2020.3047728

[28] Li Y, Wu D, Wang H, Lou J. Dynamic collision avoidance for maritime autonomous surface ships based on deep Q-network with velocity obstacle method. Ocean Eng. 2025;320:120335. doi: 10.1016/j.oceaneng.2025.120335

[29] Yang X, Lou M, Hu J, Ye H, Zhu Z, Shen H, et al. A human-like collision avoidance method for USVs based on deep reinforcement learning and velocity obstacle. Expert Syst Appl. 2024;254:124388. doi: 10.1016/j.eswa.2024.124388

[30] Everett M, Chen YF, How JP. Collision avoidance in pedestrian-rich environments with deep reinforcement learning. IEEE Access. 2021;9:10357–77. doi: 10.1109/access.2021.3050338

