# Correction: Optimized real-time path planning for micro UAVs in dynamic environments aided by reciprocal velocity obstacle algorithm

**DOI:** 10.1371/journal.pone.0345474

**Published:** 2026-03-19

**Authors:** Pengxiang Sun, Wei Sun, Wei Ding, Yadan Li, Jingang Zhao

The images for Figs 10 and 12 are incorrect. The authors have provided a corrected version of figures here.

**Fig 10:**
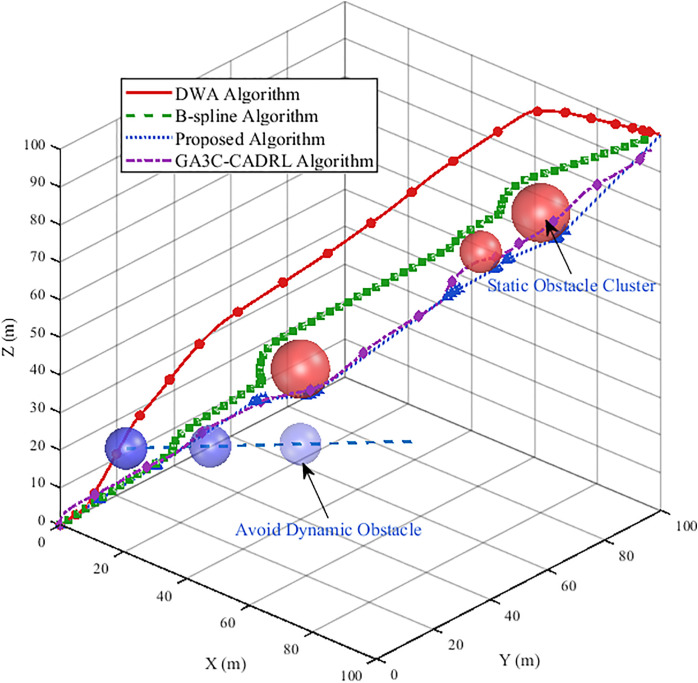
Figure title UAV trajectory diagram for simulation experiment 1.

**Fig 12:**
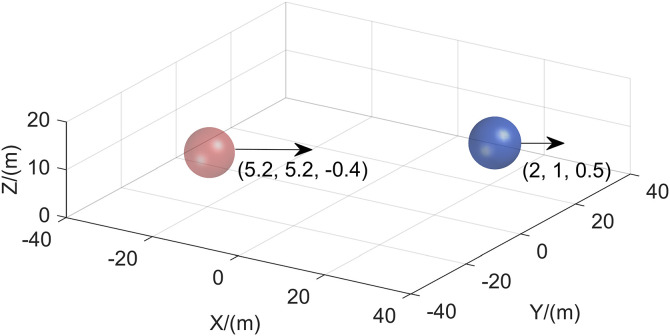
Figure title Initial environment for multi-dynamic obstacles.
